# Effectiveness of Inspiratory Muscle Training in Individuals with Chronic Venous Disease: A Randomized Controlled Study

**DOI:** 10.3390/life15020296

**Published:** 2025-02-14

**Authors:** Cemre Görünmezoğlu, Özlem Çinar Özdemir, Gülşah Barğı, Dündar Özalp Karabay

**Affiliations:** 1 Department of Physiotherapy and Rehabilitation, Faculty of Health Sciences, İzmir Democracy University, 35140 İzmir, Turkey; cemregorunmezoglu@gmail.com (C.G.); gulsah.bargi@idu.edu.tr (G.B.); 2 Department of Cardiovascular Surgery, Faculty of Medicine, Dokuz Eylül University, 35220 İzmir, Turkey; ozalp.karabay@deu.edu.tr

**Keywords:** breathing exercises, pain, quality of life, respiratory function test, 6-min walk test, venous disease

## Abstract

This study aimed to investigate the effects of 6-week inspiratory muscle training (IMT) on pain, pulmonary functions, respiratory muscle strength, lower extremity functionality, exercise capacity and quality of life (QoL) in individuals with chronic venous disease (CVD). Individuals were randomly assigned to training (TG) (*n* = 15, 45.53 ± 8.64 years) and control (CG) (*n* = 15, 47 ± 9.30 years) groups. While individuals in the TG performed IMT (30 min/everyday), individuals in the CG performed thoracic expansion exercises (TEEs) (10 respiratory cycles/set, four sets/day). Pain, pulmonary function and respiratory muscle strength tests, lower body strength and functional mobility, submaximal exercise capacity and QoL were assessed in all individuals. After 6 weeks, FEV_1_/FVC, PEF, FEF_25–75%_ and MIP significantly increased in the TG compared to the CG (*p* < 0.05). Within the TG, FEV_1_, PEF, FEF_25–75%_, MIP, sit-to-stand number and 6-MWT distance significantly increased while resting pain, activity pain and QoL scores significantly decreased after 6 weeks (*p* < 0.05). Within the CG, FVC and sit-to-stand number significantly increased while activity pain and QoL scores decreased after 6 weeks (*p* < 0.05). Inspiratory muscle strength and pulmonary functions improved following IMT compared to TEE in individuals with CVD. As pulmonary functions, pain, lower extremity functionality and QoL may improve via IMT or TEE in individuals with CVD, submaximal capacity may improve following IMT.

## 1. Introduction

Chronic venous disease (CVD) is a chronic pathological condition that describes both visual and functional manifestations of abnormalities in the peripheral venous system [1]. CVD causes heaviness, tiredness, itching of the skin, nocturnal cramps and throbbing and aching of the legs, which is exacerbated by prolonged standing [2]. Although a complete understanding of the pathophysiology of CVD remains uncertain, chronic venous hypertension is widely accepted as the predominant cause of advanced venous skin changes and ulceration. As a result of these conditions, while venous pressure increases, both blood return is impaired and venous pathology occurs [3,4]. The main causes of these pathologies are venous obstruction, valve insufficiency or muscle pump dysfunction [3]. Muscle pump dysfunction includes dysfunctions that occur in not only calf muscles but also inspiratory muscles [5]. During deep inspiration, while pressure in the abdomen increases, pressure in the thorax decreases via the descent of the diaphragm. Thus, deep inspiration increases negative intrathoracic pressure and creates a partial vacuum effect in the thorax, facilitating venous return to the inferior vena cava and right atrium [6]. Simultaneously with diaphragm contraction, the abdominal veins are compressed. These changes in intrathoracic and intra-abdominal pressures that occur throughout respiration facilitate venous return due to the obstruction of retrograde flow in the veins [7]. It has been also shown that the respiratory cycle affects venous return through the increase in deep inspiration and the flow rate of the femoral vein in healthy individuals [7,8]. When inspiratory muscle strength is insufficient, peripheral blood flow decreases, vascular resistance in the leg veins increases and, therefore, systemic venous return may decrease [9].

Inspiratory muscle training (IMT) is performed by inhaling against an external inspiratory load provided by diverse devices. Its main purpose is to strengthen the diaphragm and other accessory respiratory muscles [10]. IMT is recommended to individuals with cardiac disease, particularly those with coronary artery disease and chronic heart failure [11,12,13,14,15,16,17]. Significant improvements in submaximal exercise capacity, balance, respiratory and peripheral muscle strength, dyspnea and depression levels have occurred in individuals with chronic heart failure performing IMT [13]. Studies have also demonstrated that the inclusion of IMT with aerobic and resistive exercises improves maximal inspiratory pressure (MIP), maximal expiratory pressure (MEP), oxygen consumption and quality of life (QoL) compared to aerobic and resistive exercise alone in individuals undergoing coronary artery bypass surgery and phase II cardiac rehabilitation [14]. In studies examining the effectiveness of IMT combined with aerobic exercise training compared to aerobic exercise training alone, combined training was found to be more effective on oxygen consumption, endurance, MIP and QoL [15,16]. The IMT has been reported to improve submaximal exercise capacity due to improved systemic vasodilation and perfusion of peripheral muscles [12,17]. A newly published study found that IMT and compression therapy had a significant healing effect on disease severity and respiratory muscle strength compared to calf muscle exercise training and compression therapy in individuals with CVI [5]. Although effects of IMT on clinical outcomes have been demonstrated in many patients with cardiovascular disease, the effects of IMT have not yet been adequately investigated in individuals with CVD. Therefore, it has been hypothesized that performing regular 6-week IMT as a novel and alternate therapy for CVD may help increase venous return and reduce the symptoms of CVD in the current study. It was aimed at investigating the potential benefits, if any, of inspiratory muscle training on pain, pulmonary functions, respiratory muscle strength, lower extremity functionality, functional exercise capacity and QoL in individuals with CVD in this prospective randomized controlled study.

## 2. Materials and Methods

### 2.1. Study Design

The present study was planned as a prospective, single-blind, randomized controlled study. Individuals were randomly allocated to either the training group (TG) or the control group (CG) using a computer-based program. To perform single blinding, it was not reported which groups the individuals with CVD belonged to. The TG received IMT while CG performed thoracic expansion exercises (TEEs) for 6 weeks. The outcomes were pain, pulmonary functions, respiratory muscle strength, lower extremity functionality, functional exercise capacity and QoL. While the primary outcome was MIP, the secondary outcomes were others. All assessments were performed in in-person sessions before and after 6-week follow-up.

The study was approved by the Izmir Democracy University Non-Invasive Clinical Research Ethics Committee with decision number 2023/08-02 on 21 June 2023 and conducted in accordance with the principles of the Declaration of Helsinki. The study was carried out at the Department of Physiotherapy and Rehabilitation, Faculty of Health Sciences in Izmir Democracy University. The individuals were referred to the Physiotherapy and Rehabilitation Department from the Department of Cardiovascular Surgery, Faculty of Medicine in Dokuz Eylul University between July 2023 and May 2024. Written informed consent was received from all individuals. The study protocol was registered at ClinicalTrials.gov (NCT05993650).

### 2.2. Study Population

**Figure 1:**
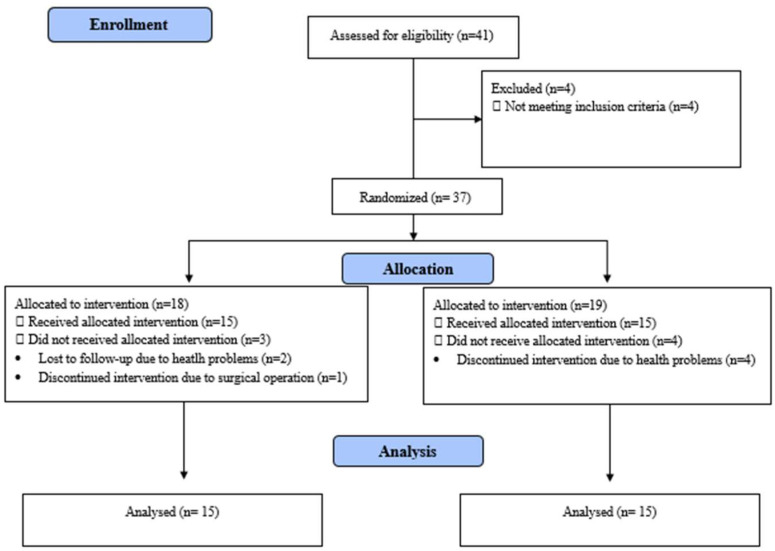
Flow diagram of the current study.

A total of 41 individuals with CVD were included. A total of 30 individuals were included since they met the inclusion criteria and volunteered for the study; 15 individuals were directed to each of two groups (Figure 1). The inclusion criteria for these individuals with CVD were 18 to 65 years of age, having a diagnosis of CVD using duplex ultrasound, having a score for Clinical, Etiologic, Anatomic, Pathophysiologic (CEAP) Classification of C1, C2, C3, C4 or C5 [18] and agreeing to participate in the study. The exclusion criteria for these individuals were having arterial diseases, cardiorespiratory diseases, acute ulcer, diabetic ulcers, a history of deep vein thrombosis and/or venous system surgery and orthopedic and neurological disorders that would affect walking or being pregnant. Individuals in both groups used medication containing the same active ingredient (calcium dobesilate) both during and after treatment.

### 2.3. Interventions

**Figure 2:**
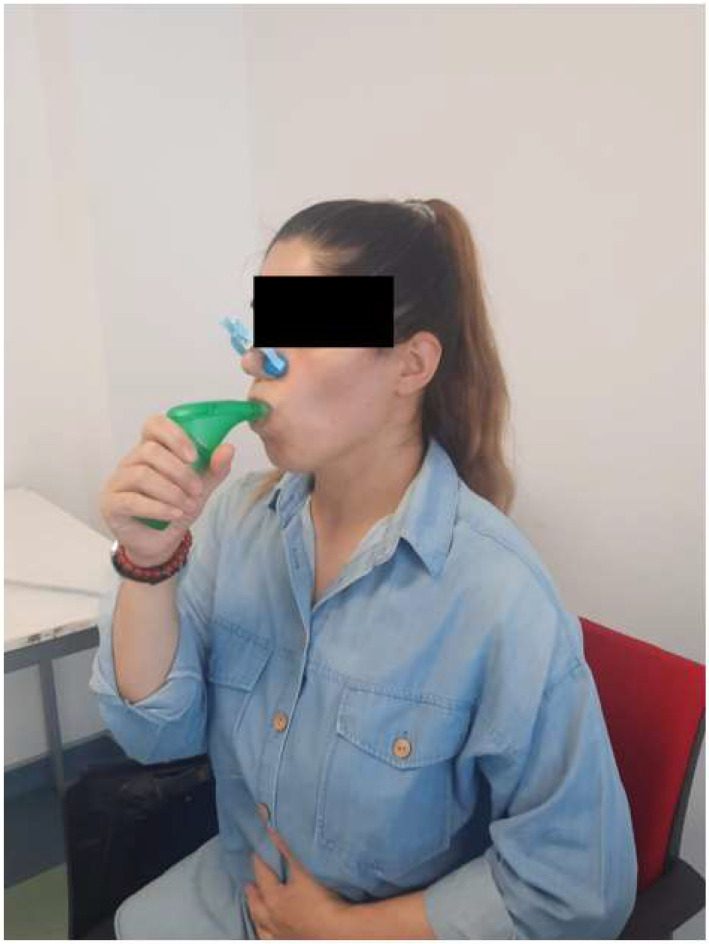
IMT application.

**Figure 3:**
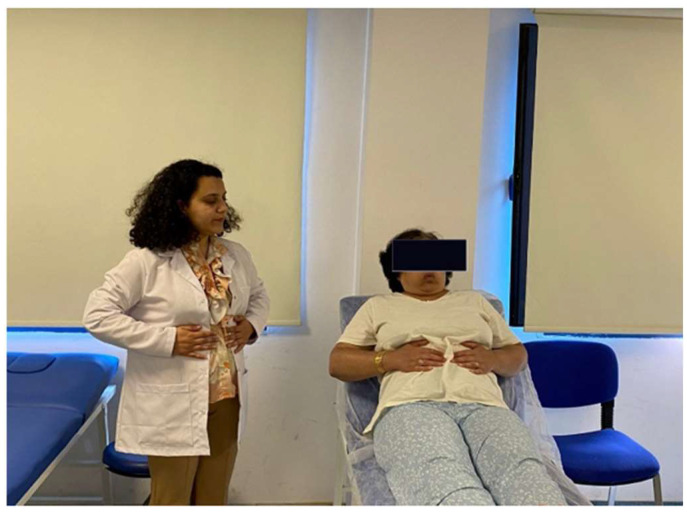
Thoracic expansion exercise.

The individuals in the TG performed IMT for 30 min/day, 7 days/week (one day of the week under the supervision of a physiotherapist and another 6 days without supervision at home) for a total of 6 weeks (Figure 2). The physiotherapist followed the exercise diary of the individuals with CVD at weekly check-ups and called people every week to check their compliance with the exercise program. The individuals in CG performed only TEEs (3 deep breathing exercises/respiratory cycle, 10 respiratory cycles/set, 4 sets/day, 7 days/week), as well (Figure 3).

Individuals in both groups were provided with the same information within the framework of patient education. In this context, individuals were advised to quit smoking, adopt a low-fat and high-fiber diet, elevate their legs above heart level for at least 30 min/day, avoid staying in a fixed position (standing, sitting, etc.) for long periods of time, avoid wearing high-heeled shoes and tight clothing, avoid hot baths and saunas, do 30 min/day of walking exercise and regularly use compression stockings recommended by the medical doctor [19].

#### 2.3.1. The IMT

This training was performed with the POWERbreathe^®^ Plus (International Ltd., Warwick, UK) device which works on the threshold loading principle. The threshold loading principle is used to inhale against the same pressure load in every inhalation for strengthening primarily respiratory muscles [20]. The IMT was performed for 30 min/day, 7 days/week and 6 weeks. One day of the week, a session was carried out under the supervision of a physiotherapist (Figure 2). Individuals’ heart rate, respiratory rate and oxygen saturation were monitored during supervised sessions. The other 6 days of the week, sessions were practiced without supervision at home, which was planned as a home program. In the first supervised session, individuals with CVD were taught the correct and effective use of this device by taking sufficient diaphragmatic breaths. Initial training intensity was adjusted at 30% of the initial MIP measurement of the individuals. Each week, MIP values of the individuals were measured and a new training intensity for IMT was determined each week. The new training load was applied by gradually increasing it to 30–50% of the measured new MIP value. The training load of the individuals was adjusted to the appropriate level on the device based on the new MIP value measured every week. With the new training load, the first session of each week was held under the supervision of the physiotherapist. During the application of IMT, individuals were asked to sit in an upright position with the upper chest and shoulders in a relaxed position. After the nose clip was attached, the individuals were asked to take a deep breath by closing their lips tightly and exhaling by blowing out slowly. Individuals were instructed to maintain a breathing set which consisted of consecutive 10–15 diaphragmatic breathings and 4–5 calm breaths resting following the set. Individuals maintained these breathing sets for 30 min/day. The individuals were trained for 7 days/week, 6 weeks in total [21]. Daily record charts were required and reviewed to emphasize training control. Regular calibration, monitoring and device control occurred at specified intervals. Individuals were asked not to change the intensity of training during the unsupervised period at home and not to engage in exercise or physical activity outside of their normal routine. The total time spent on IMT recorded in individuals’ diaries was calculated in minutes as the total workload for everyone.

#### 2.3.2. The TEE

The TEEs are deep breathing exercises that emphasize deep inspiration and controlled expiration [22]. The TEEs were performed in the upright sitting position (Figure 3). In this position, the individuals were asked to place their hands on their lower costae and then do the cycle of ‘deep breathing + holding the deep breath for 3 s + slowly releasing the entire breath’ 3 times in a row. After this cycle, individuals were instructed to rest by breathing calmly three or four times. Immediately afterwards, the individuals were asked to repeat the same cycle 10 consecutive times [22] and perform this session 4 times a day and every day for 6 weeks. Individuals performed the first session of TEEs under supervision and completed the remaining sessions as part of a home exercise program. Individuals in the CG were also called once a week by phone to check whether they were adhering to the exercise program.

### 2.4. Measurements

The individuals’ demographic data and outcome measurements including pain, pulmonary functions, respiratory muscle strength, lower body strength and functional mobility, submaximal exercise capacity and QoL were recorded using an evaluation form.

#### 2.4.1. Pain

Individuals were first asked about pain localization in the lower extremity which was recorded in the evaluation form. Then, pain severity was measured using the Numeric Rating Scale (NRS). The maximum pain intensity at rest and activity was scored from 0 (no pain) to 10 (worst pain) [23]. For pain at rest, we assessed “stimulus-independent” pain by inquiring whether patients experienced pain without any exertion or physical movement. For pain at activity, we evaluated the presence of pain that commonly occurs during normal activities (e.g., coughing or walking), specifically pain that is triggered or intensified by movement or stimulation.

#### 2.4.2. Pulmonary Functions and Respiratory Muscle Strength

The tests for pulmonary function and respiratory muscle strength were performed using a spirometry instrument (Cosmed Pony FX, Rome, Italy) [24,25]. The individuals rested for 15 min before undergoing the pulmonary function test. While the individuals were sitting in a comfortable position on a backed chair, a nose clip was placed on their noses, and they were asked to bite the mouthpiece with their teeth and close their lips in a way that would not allow air to escape. During the test, individuals were asked to exhale until there was no air left in their lungs. The tests were performed at least 3 times [24]. Dynamic lung volumes including forced expiratory volume in the first second (FEV_1_), forced vital capacity (FVC), forced expiratory volume in the first second/forced vital capacity (FEV_1_/FVC), peak expiratory flow (PEF) and forced expiratory flow from 25% to 75% (FEF_25–75%_) were measured and expressed as percentages of the expected values according to age, height, body weight and gender [24,26].

The MIP and MEP were recorded during a few seconds of maximal oral inspiration (Müller maneuver) and maximal oral expiration (Valsalva maneuver), respectively. The MIP was measured by making individuals take a rapid and deep inspiration at the residual volume immediately after their maximum expiration. The MEP was measured by making individuals take a maximum inspiration followed by a rapid and deep exhalation at total lung capacity. Tests were performed in a sitting position using a nose clip. Individuals were verbally encouraged for the best measurement result. The measurement was repeated in the individuals until a valid value was obtained. If there was more than 5% or 5 cmH_2_O difference between consecutive measured values, the measurement was repeated. The highest value among the measurements was selected for statistical analyzis [25]. Reference equations for the MIP and MEP normal values were used to interpret the measurements [27]. The minimal clinically important difference is 13 cmH_2_O for inspiratory muscle strength [28].

#### 2.4.3. Lower Body Strength and Functional Mobility

Functional mobility was evaluated using a 30 s chair stand test [29]. First, individuals sit on a chair with back support and no arm support, with their hands crossed on the shoulders. Then individuals were asked to stand and sit down after standing in an upright position. Individuals were asked to repeat the sit-to-stand movement as fast as possible for 30 s. Before beginning the test, the physiotherapist also demonstrated how to perform the sit-to-stand movement properly. After the individual had given the ‘Start’ command, stopwatch time was started. The number of correct repetitions of the movement within 30 s was recorded. To maintain physical independence, cut off scores were stated as 15 repetitions for women and 17 repetitions for men [30].

#### 2.4.4. Submaximal Exercise Capacity

Submaximal exercise capacity was evaluated using the 6 min walk test (6-MWT) according to American Thoracic Society criteria [31]. The 6-MWT was applied twice on the same day, within a half hour interval. Individuals were asked to walk as fast as they could in a 30 m straight corridor for 6 min at their own walking pace without running. To encourage the individuals, standard expressions were used every minute during the test and the 6-MWT distance was recorded in meters. Heart rate, respiratory rate, oxygen saturation, blood pressure, fatigue and dyspnea perceptions were recorded before and after 6-MWT. The highest distance was recorded and expressed as the percentage of the predicted values [32]. The minimal clinically important difference is 25 m for 6-MWT [33].

#### 2.4.5. The QoL

The Chronic Venous Disease QoL Questionnaire (CIVIQ-20) was used to evaluate the QoL in individuals with CVD [34]. The Turkish version of this questionnaire is valid and reliable [35]. The CIVIQ-20 comprises 20 items and is divided into four dimensions (physical, psychological, social status and pain). Each item is scored on a 5-item Likert scale; 1 represents no pain or impairment while 5 indicates severe pain or impairment. The total score varies from 0 (highest QoL) to 100 (lowest QoL) [34].

### 2.5. Statistical Analyzes

Sample size analysis was performed before conducting the study. To determine mean difference in MIP scores between two independent groups [5] for an α value of 0.05 and a power of 95%, at least 8 individuals in the TG and 8 individuals in the CG were calculated through the GPower program (G*Power 3.0.10 system, Franz Faul, Universität Kiel, Kiel, Germany). Considering the possibility of loss, a plan was made to have at least 15 individuals in each group. Statistical analyzes were performed using SPSS 25.0 software (IBM Corp., Armonk, NY, USA) after data collection [36]. The conformity of variables to a normal distribution was investigated using both visual methods (histogram and probability graphs) and the Shapiro–Wilk test. Descriptive analyzes were provided as frequency (n) and percentage (%) values for the categorical variables and mean (x) and standard deviation (sd) values for the normally distributed variables. Chi-square and Fisher tests were used to compare categorical variables of the groups, respectively. The intra-group and inter-group (delta (Δ) changes) comparisons of pre- and post-training variables showing a normal distribution were made using the paired samples *t*-test and independent samples *t*-test, respectively. The probability of error in statistical analysis was set as *p* < 0.05.

## 3. Results

Among the 41 individuals with CVD, 15 in the TG and 15 in the CG completed the follow-up (Figure 1). The evaluations of all individuals in the groups were performed face-to-face. The demographic and clinical characteristics of the groups were similar and presented in Table 1 (*p* > 0.05). Baseline values for respiratory muscle strength, pain, pulmonary functions, lower body strength and functional mobility, submaximal exercise capacity and QoL of the groups before training were also similar (Table 1 and Table 2, *p* > 0.05). A total of 15 (100%) individuals with CVD in the TG and 13 (86.7%) in the CG had resting pain while 13 (86.7%) in the TG and 15 (100%) in the CG had pain at activity at baseline (*p* > 0.05). Only 1 (6.7%) person in the TG and 1 (6.7%) in the CG could maintain physical independence according to cut off scores for the 30 s chair stand test at baseline (*p* > 0.05).

A comparison of the values within the groups before and after exercise training is presented in Table 2. While the baseline values of MIP, FEV_1_, PEF, FEF_25–75%_, sit-to-stand number and 6-MWT distance increased statistically significantly after 6 weeks within the TG, resting pain, activity pain and QoL scores decreased statistically significantly after 6 weeks within the TG (Table 2, *p* < 0.05). The baseline values of FVC and sit-to-stand number values increased significantly after 6 weeks within the CG, while activity pain and QoL scores significantly decreased after 6 weeks within the CG (Table 2, *p* < 0.05).

Comparisons of the intra-group difference values between the groups before and after exercise training are presented in Table 3. After 6 weeks, the ΔMIP, ΔFEV_1_/FVC, ΔPEF and ΔFEF_25–75%_ values increased statistically significantly in the TG compared to the CG (Table 3, *p* < 0.05). Moreover, after 6 weeks, 11 (73.3%) in the TG and 2 (13.3%) in the CG reached 13 cmH_2_O difference for MIP increase (*p* < 0.05) and 8 (57.1%) in the TG and 3 (21.4%) in the CG reached 25 m difference for 6-MWT increase (*p* > 0.05). No adverse event or complication developed in individuals with CVD following IMT and TEE.

## 4. Discussion

This prospective randomized controlled study indicated that IMT provides significant benefits for the health of individuals with CVD through improving pulmonary functions and inspiratory muscle strength compared to TEE. On the other hand, when individuals with CVD perform only IMT, pain, pulmonary functions, inspiratory muscle strength, lower extremity functionality, submaximal exercise capacity and QoL may improve after 6 weeks of training. If individuals with CVD perform only TEE, activity pain, FVC value, lower extremity functionality and QoL may improve after 6 weeks. As a safe new method, IMT both has considerable effects on pulmonary functions and inspiratory muscle strength and improves pain, lower extremity functionality, exercise capacity and QoL in individuals with CVD.

Current physiotherapy approaches in managing CVD focus primarily on enhancing venous return and improving overall patient quality of life. For these purposes, exercise training is increasingly recognized as an adjunctive therapy for CVD. Regular physical activity, particularly exercises targeting the muscle pump, can enhance venous return and mitigate symptoms of CVD [37].

It is important to strengthen the inspiratory muscles since both healthy individuals and individuals with various clinical disorders can experience various physical limitations due to respiratory muscles weakness [11,12]. The curative effect of IMT on symptoms and its safety as a device in the clinical follow-up and rehabilitation of various diseases have been demonstrated in many studies [5,10,11,12,13,14,15,16,17]. To the best of our knowledge, one previous study has investigated the effectiveness of IMT in individuals with CVI [5]. Aydın et al. (2022) investigated the effects of IMT and calf muscle exercise training (CMET) applied in addition to compression therapy on QoL, venous filling time, disease severity, pain, edema, range of motion, muscle strength and functionality [5]. In the study, Group 1 received IMT and compression therapy, Group 2 received CMET and compression therapy, while Group 3 received only compression therapy [5]. As a result, group 2 showed more improvement in terms of QoL, venous filling time, pain, edema, range of motion, muscle strength and functionality compared to other groups. Group 1 showed more improvement in disease severity and inspiratory and expiratory muscle strength values than groups 2 and 3 while only physical mobility and right leg venous filling time increased in group 3 [5]. Moreover, this study has revealed that enhanced inspiratory muscle power is directly associated with improved venous refilling time and functional capacity [5]. The study has also presented a comparative analysis of IMT and CMET, highlighting their respective impacts on quality of life, venous function and pain management [5]. Like the results of this study [5], significant increases in inspiratory muscle strength were obtained with IMT compared to the control group in our study. On the other hand, significant improvements in pain and QoL following both IMT and TEE groups were obtained in current study in contrast to results of the study conducted by Aydın et al. (2022). In addition, submaximal exercise capacity improved through only IMT in our study. These significant improvements following IMT application on physical activity level, peripheral muscle strength, lung function, exercise capacity and QoL have been demonstrated in patients with hypertension, as well [38].

Studies show that as the clinical severity of chronic venous disease increases, the QoL of individuals decreases [39]. The results of studies presenting the effects of various exercise training on QoL in individuals with chronic venous disease are contradictory. The QoL may not improve following strengthening exercises alone [40] or combined aerobic and strengthening exercises [41]. However, Leal et al. [42] and Gürdal Karakelle et al. [43] showed significant improvements in QoL following strengthening activities. In parallel with the results of these studies [42,43], our patients’ disease-specific QoL scores were improved after either IMT or TEE. Dominelli et al. [44] showed that when the respiratory workload decreases during exercise, the blood flow to the respiratory muscles decreases but blood flow to the peripheral muscle increases, and vice versa. These changes in leg blood flow due to the work of breathing indicate a competitive relationship between the locomotor and respiratory muscles for a limited cardiac output. The physiological basis of this phenomenon is that high levels of respiratory muscle activation during exercise and the resulting metabolite accumulation in the respiratory muscles cause reflex vasoconstriction in the vessels of the extremity muscles [44,45]. Similarly, the significant improvements in QoL and lower extremity functionality after IMT or TEEs demonstrated in the present study may have resulted from increased blood flow to the lower extremities through increased respiratory muscle strength and reduced respiratory workload.

Dogru-Huzmeli et al. [46] showed that mobility and diaphragmatic breathing exercises performed as a part of dance therapy have improved pain and QoL in patients with CVI. Naci et al. [47] reported that calf muscle pumping, flexibility and diaphragmatic breathing exercises performed in addition to compression therapy have decreased pain in patients with CVI. Although different breathing exercises were used in our study than in these studies, similar improvements in terms of pain and QoL were observed in our study. Also, 100% individuals with CVD in the TG and 86.7% in the CG had resting pain while 86.7% in the TG and 100% in the CG had pain on movement at baseline in the current study. Our findings are comparatively higher in contrast to the prevalence of pain in CVD reported in the recent literature. In the study by Bradbury et al. [48], 45% of women without varicose veins and 63% of those with grade 2 and 3 varicose veins had pain, while in the study by Vuylsteke et al. [49], which included 6009 male and female patients, the prevalence of pain was reported as 54%. Jawien et al. [50] reported that 81% of CVI patients with varicose veins had leg pain. Contrary to the current study, these studies were conducted with much larger samples. Most of the individuals who participated in our study had a sedentary lifestyle and high BMI values. In addition, the subjective assessment of pain could be the factor contributing to the comparatively higher prevalence of pain in this study. Additionally, the subjective pain perception of patients suffering from chronic disease may also have increased over time. As previously reported by Chiappa et al. [17], IMT has a significant effect on functional capacity by improving systemic vasodilatation and perfusion of peripheral muscles in patients with chronic heart failure in agreement with our result. However, Albuquerque et al. [51] showed that 6 weeks of IMT resulted in improvements in inspiratory muscle strength but failed to produce significant improvements in functional capacity. Similarly, Mills et al. [52] noted an increase in inspiratory muscle strength via IMT in older adults, but no increase in submaximal exercise capacity was observed. In our study, while a significant increase in lower extremity functionality and submaximal exercise capacity was observed after IMT, only an increase in lower extremity functionality was observed after TEE. This is contrary to the results of these studies regarding functional capacity [51,52]. A possible explanation for this difference in submaximal exercise capacity found in studies on elderly individuals and in our study may be that the individuals in our study were younger than 65 years of age. Moreover, stronger performance of the respiratory muscle pump resulted in significant reductions in pain, lower extremity functionality, submaximal exercise capacity and QoL. Our findings, therefore, suggest that IMT could be an effective treatment option for improving pain, pulmonary functions, inspiratory muscle strength, lower extremity functionality, submaximal exercise capacity and QoL in patients with CVD.

The present study has some limitations. The first limitation of the study is that exercise capacity could not be evaluated with CPET. Although CPET is the gold standard for assessing exercise capacity, we assessed exercise capacity with a practical field test, the 6-MWT, as we did not have this equipment at our institution. The second limitation of our study is that the long-term effects of the IMT have not yet been investigated due to lack of time.

## 5. Conclusions

The use of either IMT or TEEs was found to be effective on pain, pulmonary function, lower extremity functionality, and QoL in individuals with CVD in the current study. On the other hand, IMT provided significant benefits in improving pulmonary functions and inspiratory muscle strength of the individuals with CVD compared to TEE applied without resistance to breathing. These findings suggest that IMT can be used as an effective treatment for individuals with CVD. In clinical settings, when the devices used for IMT are not available, TEEs can also be provided to individuals to improve their functionality and relieve their symptoms. We recommend that IMT be added to the rehabilitation programs of patients with CVD since it has an important role in supporting respiratory functions. In addition, this study demonstrated the effectiveness of moderate-intensity IMT compared to sham-intensity TEE; there is still a need to investigate the effectiveness of inspiratory muscle training applied at different intensities in patients with chronic venous disease.

## Figures and Tables

**Table 1:** Demographic and clinical characteristics of individuals with CVD.

Variables		TG (*n* = 15)	CG (*n* = 15)	*p* Value
		x ± sd	x ± sd	
Age (years)		45.53 ± 8.64	47.00 ± 9.30	0.658
BMI (kg/m^2^)		29.72 ± 4.84	29.76 ± 5.41	0.983
Number of pregnancies		1.60 ± 1.29	2.47 ± 1.85	0.148
Duration of illness (year)		7.48 ± 7.49	6.34 ± 9.02	0.710
Smoking exposure (package × year)		19.25 ± 14.10	22.00 ± 2.83	0.809
MIP (cmH_2_O)		100.20 ± 28.50	82.60 ± 31.08	0.117
MIP (%)		119.10 ± 32.56	100.55 ± 38.28	0.164
Pain at rest (NRS, 0–10)		5.33 ± 2.29	5.33 ± 3.02	1.000
Pain at activity (NRS, 0–10)		4.53 ± 2.90	6.20 ± 3.45	0.163
CIVIQ-20 score (0–100)		56.40 ± 14.95	58.27 ± 13.35	0.721
		*n*; %	*n*; %	
Gender	Female	12; 80%	13; 86.7%	1.000
	Male	3; 20%	2; 13.3%	
CEAP score	C2	5; 33.3%	6; 40%	0.494
	C3	7; 46.7%	4; 26.7%	
	C4	3; 20%	5; 33.3%	
Previous history of COVID-19	Yes	7; 46.7%	8; 53.3%	0.715
	No	8; 53.3%	7; 46.7%	
Affected extremity	Right	1; 6.7%	3; 20%	0.136
	Left	1; 6.7%	4; 26.7%	
	Bilateral	13; 86.6%	8; 53.3%	
Smoking status	Yes	4; 26.7%	1; 6.7%	0.227
	No	11; 73.3%	13; 86.7%	
	Ex-smoker	0; 0%	1; 6.7%	
Alcohol consumption status	Yes	1; 6.7%	0; 0%	0.343
	No	14; 93.3%	15; 100%	
Presence of CVD in the family	Yes	5; 33.3%	8; 53.3%	0.269
	No	10; 66.7%	7; 46.7%	
Usage of compression stockings	Yes	6; 40%	3; 20%	0.427
	No	9; 60%	12; 80%	

m: meter, kg: kilogram, BMI: body mass index; n: frequency, %: percentage, MIP: maximal inspiratory pressure, NRS: Numeric Rating Scale, CIVIQ-20: Chronic Venous Disease Quality of Life Questionnaire, CEAP: Clinical, Etiologic, Anatomic, and Pathophysiologic classification, COVID-19: SARS-CoV-2, CVD: chronic venous disease, TG: training group, CG: control group, x: mean, sd: standard deviation, *p*: *p* value. Student—*t* Test *p* < 0.05, Chi-Square Test *p* < 0.05.

**Table 2:** Comparison of measurement values within the groups before and after 6-week follow-up.

Variables	TG (*n* = 15)			CG (*n* = 15)		
	Before	After	Within-Groups *p* Value	Before	After	Within-Groups *p* Value
	x ± sd	x ± sd		x ± sd	x ± sd	
MIP (cmH_2_O)	100.20 ± 28.50	128.73 ± 23.96	0.001 *	82.60 ± 31.08	81.93 ± 27.77	0.857
MIP (%)	119.10 ± 32.56	153.24 ± 27.76	0.001 *	100.55 ± 38.28	99.66 ± 32.93	0.854
MEP (cmH_2_O)	109.53 ± 38.44	119.73 ± 34.67	0.148	93.33 ± 31.22	95.00 ± 34.59	0.616
MEP (%)	111.43 ± 44.76	120.06 ± 32.93	0.238	98.53 ± 35.97	99.87 ± 38.95	0.720
Pain at rest (NRS, 0–10)	5.33 ± 2.29	3.13 ± 2.97	0.026 *	5.33 ± 3.02	4.00 ± 2.48	0.094
Pain at activity (NRS, 0–10)	4.53 ± 2.90	2.47 ± 2.36	0.032 *	6.20 ± 3.45	4.60 ± 3.48	0.031 *
FEV_1_ (L)	2.54 ± 0.84	2.77 ± 0.74	0.036 *	2.31 ± 0.66	2.33 ± 0.72	0.613
FEV_1_ (%)	81.67 ± 18.51	89.80 ± 12.74	0.095	87.53 ± 15.94	87.80 ± 16.46	0.896
FVC (L)	3.54 ± 1.10	3.57 ± 0.99	0.535	3.12 ± 0.90	3.22 ± 0.90	0.061
FVC (%)	96.00 ± 15.33	98.07 ± 12.57	0.498	99.00 ± 10.57	102.53 ± 10.71	0.041 *
FEV_1_/FVC (%)	71.98 ± 12.34	77.99 ± 4.97	0.056	74.07 ± 9.64	72.78 ± 9.36	0.396
PEF (L)	3.97 ± 1.63	4.98 ± 2.09	0.013 *	4.03 ± 1.82	4.11 ± 2.35	0.822
PEF (%)	53.93 ± 19.96	67.93 ± 21.78	0.009 *	61.20 ± 25.84	61.00 ± 30.06	0.967
FEF_25–75%_ (L)	2.31 ± 1.03	2.65 ± 0.93	0.044 *	2.24 ± 0.68	2.09 ± 0.69	0.250
FEF_25–75%_ (%)	62.80 ± 23.98	71.00 ± 18.58	0.106	65.33 ± 17.10	61.27 ± 18.18	0.284
30 s chair stand test (n)	11.00 ± 2.98	13.13 ± 3.34	0.014 *	9.87 ± 2.13	11.27 ± 1.91	0.011 *
6-MWT (m)	475.38 ± 67.58	497.65 ± 71.19	0.035 *	466.19 ± 80.56	466.06 ± 75.56	0.989
6-MWT (%)	83.49 ± 9.31	87.45 ± 10.30	0.033 *	82.41 ± 11.72	82.69 ± 13.05	0.870
CIVIQ-20 score (0–100)	56.40 ± 14.95	40.67 ± 14.74	<0.001 *	58.27 ± 13.35	47.13 ± 14.92	<0.001 *

NRS: Numeric Rating Scale, FEV_1_: forced expiratory volume exhaled in the first second, FVC: forced vital capacity, PEF: peak expiratory flow, FEF_25–75%_: forced expiratory flow from 25% to 75%, MIP: maximal inspiratory pressure, MEP: maximal expiratory pressure, s: second, 6-MWT: 6 min walk test, CIVIQ-20: Chronic Venous Disease Quality of Life Questionnaire, L: liter, %: percentage, cmH_2_O: centimeter of water, n: frequency, m: meter, TG: training group, CG: control group, x: mean, sd: standard deviation, *p*: *p* value. Paired samples *t*-test, * *p* < 0.05.

**Table 3:** Comparison of the intra-group difference values between the groups before and after exercise training.

	Intra-Group Difference (Δ) Values		Intergroup Difference Values	
	TG (*n* = 15)	CG (*n* = 15)		
	x ± sd	x ± sd	Mean Difference (95%CI)	*p* Value
MIP (cmH_2_O)	28.53 ± 27.52	−0.67 ± 14.11	29.20 [(12.59)–(45.81)]	0.001 *
MIP (%)	34.14 ± 33.14	−0.90 ± 18.50	35.04 [(14.72)–(55.36)]	0.002 *
MEP (cmH_2_O)	10.20 ± 25.81	1.67 ± 12.59	8.53 [(−6.92)–(23.99)]	0.263
MEP (%)	8.63 ± 27.11	1.34 ± 14.20	7.29 [(−9.14)–(23.71)]	0.367
Pain at rest (NRS, 0–10)	−2.20 ± 3.43	−1.33 ± 2.87	−0.87 [(−3.23)–(1.50)]	0.459
Pain at activity (NRS, 0–10)	−2.07 ± 3.37	−1.60 ± 2.59	−0.47 [(−2.71)–(1.78)]	0.674
FEV_1_ (L)	0.23 ± 0.39	0.02 ± 0.18	0.21 [(−0.02)–(0.44)]	0.073
FEV_1_ (%)	8.13 ± 17.57	0.27 ± 7.74	7.87 [(−2.50)–(18.24)]	0.129
FVC (L)	0.03 ± 0.18	0.11 ± 0.20	−0.08 [(−0.22)–(0.07)]	0.284
FVC (%)	2.07 ± 11.50	3.53 ± 6.08	−1.47 [(−8.35)–(5.41)]	0.666
FEV_1_/FVC (%)	6.01 ± 11.16	−1.29 ± 5.72	7.30 [(0.67)–(13.94)]	0.032 *
PEF (L)	1.01 ± 1.38	0.08 ± 1.26	0.93 [(−0.06)–(1.92)]	0.064
PEF (%)	14.00 ± 17.74	−0.20 ± 18.14	14.20 [(0.78)–(27.62)]	0.039 *
FEF_25–75%_ (L)	0.34 ± 0.59	−0.16 ± 0.50	0.50 [(0.08)–(0.91)]	0.020 *
FEF_25–75%_ (%)	8.20 ± 18.37	−4.07 ± 14.14	12.27 [(0.01)–(24.53)]	0.050 *
30-s chair stand test (n)	2.13 ± 2.95	1.40 ± 1.84	0.73 [(−1.11)–(2.57)]	0.421
6-MWT (m)	22.27 ± 35.48	−0.14 ± 36.57	22.41 [(−5.58)–(50.40)]	0.112
6-MWT (%)	3.97 ± 6.23	0.28 ± 6.36	3.68 [(−1.21)–(8.57)]	0.134
CIVIQ-20 score (0–100)	−15.73 ± 12.94	−11.13 ± 7.87	−4.60 [(−12.69)–(3.49)]	0.251

NRS: Numeric Rating Scale, FEV_1_: forced expiratory volume exhaled in the first second, FVC: forced vital capacity, PEF: peak expiratory flow, FEF_25–75%_: forced expiratory flow from 25% to 75%, MIP: maximal inspiratory pressure, MEP: maximal expiratory pressure, s: second, 6-MWT: 6 min walk test, CIVIQ-20: Chronic Venous Disease Quality of Life Questionnaire, L: liter, %: percentage, cmH_2_O: centimeter of water, n: frequency, m: meter, Δ: delta changes, TG: training group, CG: control group, x: mean, sd: standard deviation, CI: confidence interval, *p*: *p* value. Student *t* test * *p* < 0.05.

## Data Availability

The data presented in this study are available from the corresponding author upon reasonable request.
